# O.2.1-5 Knowledge, use and confidence in physical activity guidelines and assessment in recently graduated doctors from Irish medical schools: a cross sectional survey

**DOI:** 10.1093/eurpub/ckad133.110

**Published:** 2023-09-11

**Authors:** Jennifer Ryan, Dhruv Jivan, Suzanne McDonough

**Affiliations:** Royal College of Surgeons in Ireland, Ireland; suzannemcdonough@rcsi.com; Royal College of Surgeons in Ireland, Ireland; suzannemcdonough@rcsi.com; Royal College of Surgeons in Ireland, Ireland; suzannemcdonough@rcsi.com

## Abstract

**Purpose:**

Approximately 54% of the adult population in Ireland do not meet National Physical Activity Guidelines. Doctors play a key role in supporting people to be more active but may lack knowledge, skills and confidence to promote physical activity. The aim of this study was to explore knowledge and confidence in relation to physical activity counselling in recent medical graduates in Ireland.

**Methods:**

Medical doctors who graduated from Irish medical programmes in the past 5 years and currently had patient contact were asked to complete an online survey between April and November 2022. The survey was shared with medical graduates through social media, alumni groups, and relevant professional and social networks. The survey included a minimal number of questions about participant demographics and education, questions relating to participants' knowledge and confidence of physical activity guidelines and counselling, and questions relating to education and training on physical activity. The responses were downloaded to Excel and data such as mean, standard deviation, frequency and percentage, were analyzed to summarize participant responses.

**Results:**

Among the 95 responses, the majority were from graduates of Direct Entry Medicine Programmes who were currently working as Senior House Officers in either Ireland or Australia. Key findings indicated that 76% of participants were unaware of the Irish Physical Activity Guidelines and 60.76% are unaware of the Making Every Contact Count Framework. The majority wanted to receive training on physical activity at undergraduate level (73%) and postgraduate level (61%). With regards to physical activity knowledge for counselling, a majority of responders rated their knowledge between 5,6 and 7 out of 10. Similarly, a majority of responders rated their confidence in prescribing between 4,5, and 6 out of 10 (1 being not confident and 10 being extremely confident). The top three subjects participants wanted training on were: Physical Activity Guidelines, Counselling, and Assessment.

**Conclusions:**

Findings indicate a gap in the teaching and assessment of physical activity in undergraduate medical curricula in Ireland. However, there is demand among recent graduates for training on physical activity. Future studies should be done among a larger sample in order to strengthen confidence in our findings.

